# End-tidal Carbon Dioxide + Return of Spontaneous Circulation After Cardiac Arrest (RACA) Score to Predict Outcomes After Out-of-hospital Cardiac Arrest

**DOI:** 10.5811/westjem.59005

**Published:** 2023-04-04

**Authors:** Cheng-Yi Wu, Tsung-Chien Lu, Pei-I Su, Chu-Lin Tsai, Joyce Tay, Meng-Che Wu, Yi-Hsuan Yen, Eric H Chou, Chih-Hung Wang, Chien-Hua Huang, Wen-Jone Chen

**Affiliations:** * National Taiwan University Hospital, Department of Emergency Medicine, Taipei, Taiwan; † National Taiwan University, College of Medicine, Department of Emergency Medicine, Taipei, Taiwan; ‡ Baylor Scott and White All Saints Medical Center, Department of Emergency Medicine, Fort Worth, TX; § Baylor University Medical Center, Department of Emergency Medicine, Dallas, TX; || National Taiwan University Hospital and National Taiwan University College of Medicine, Department of Internal Medicine, Division of Cardiology, Taipei, Taiwan; # Min-Shen General Hospital, Taoyuan, Taiwan

## Abstract

**Introduction:**

The return of spontaneous circulation after cardiac arrest (RACA) score is a well-validated model for estimating the probability of return of spontaneous circulation (ROSC) in patients with out-of-hospital cardiac arrest (OHCA) by incorporating several variables, including gender, age, arrest aetiology, witness status, arrest location, initial cardiac rhythms, bystander cardiopulmonary resuscitation (CPR), and emergency medical services (EMS) arrival time. The RACA score was initially designed for comparisons between different EMS systems by standardising ROSC rates. End-tidal carbon dioxide (EtCO_2_) is a quality indicator of CPR. We aimed to improve the performance of the RACA score by adding minimum EtCO_2_ measured during CPR to develop the EtCO_2_ + RACA score for OHCA patients transported to an emergency department (ED).

**Methods:**

This was a retrospective analysis using prospectively collected data for OHCA patients resuscitated at an ED during 2015–2020. Adult patients with advanced airways inserted and available EtCO_2_ measurements were included. We used the EtCO_2_ values recorded in the ED for analysis. The primary outcome was ROSC. In the derivation cohort, we used multivariable logistic regression to develop the model. In the temporally split validation cohort, we assessed the discriminative performance of the EtCO_2_ + RACA score by the area under the receiver operating characteristic curve (AUC) and compared it with the RACA score using the DeLong test.

**Results:**

There were 530 and 228 patients in the derivation and validation cohorts, respectively. The median measurements of EtCO_2_ were 8.0 times (interquartile range [IQR] 3.0–12.0 times), with the median minimum EtCO_2_ of 15.5 millimeters of mercury (mm Hg) (IQR 8.0–26.0 mm Hg). The median RACA score was 36.4% (IQR 28.9–48.0%), and a total of 393 patients (51.8%) achieved ROSC. The EtCO_2_ + RACA score was validated with good discriminative performance (AUC, 0.82, 95% CI 0.77–0.88), outperforming the RACA score (AUC, 0.71, 95% CI 0.65–0.78) (DeLong test: P < 0.001).

**Conclusion:**

The EtCO_2_ + RACA score may facilitate the decision-making process regarding allocations of medical resources in EDs for OHCA resuscitation.

## INTRODUCTION

The global incidence of out-of-hospital cardiac arrest (OHCA) is estimated to be 28–44 people per 100,000 population annually.^1^ In Asia, according to the Pan-Asian Resuscitation Outcomes Study (PAROS) registry, only 5.4% of patients survived to hospital discharge and 2.7% of patients were able to recover favourable neurological function after OHCA.^2^

The concept of “chain of survival”^3^,^4^ has been proposed to streamline OHCA management to improve outcomes. With the advancement of resuscitation skills and equipment, most time-sensitive interventions can now be performed by emergency medical services (EMS) personnel in prehospital settings in a timely manner without being postponed until patients are transported to the emergency department (ED). To further improve outcomes, emergency physicians (EP) may initiate certain invasive interventions,^5^ such as extracorporeal cardiopulmonary resuscitation (CPR), for selected patients.

Accurate estimation for probability of return of spontaneous circulation (ROSC) is important for EPs in their decision-making regarding mobilising medical resources for these selected patients. The RACA^6^ score was developed to estimate the ROSC probability after OHCA and is composed of several variables, including gender, age, arrest aetiology, witness status, arrest location, initial cardiac rhythms, bystander CPR, and EMS arrival time. Nonetheless, the RACA score was initially designed for comparisons between different EMS systems by standardising ROSC rates rather than for predicting ROSC probabilities of individual patients.

End-tidal carbon dioxide (EtCO_2_) refers to the concentration of carbon dioxide at the end of exhalation, which is determined by pulmonary blood flow generated during CPR,^7^,^8^ and is suggested to be maintained above at least 10 millimetres of mercury (mm Hg) to increase ROSC probability.^9^,^10^ Therefore, in the current study we attempted to develop the EtCO_2_ + RACA score by combining the RACA score, a baseline risk-stratifying model, and minimum EtCO_2_ (a CPR quality indicator) to provide EPs with an accurate estimated ROSC probability of OHCA patients sent to the ED for ongoing CPR.

## METHODS

We performed this study by retrospectively analysing a registry database, which prospectively collected data of OHCA patients sent to the ED of the National Taiwan University Hospital (NTUH) for resuscitation. The results are reported according to the Transparent Reporting of a Multivariable Prediction Model for Individual Prognosis or Diagnosis (TRIPOD) statement.^11^

### Ethical Statements

This study was conducted in accordance with the Declaration of Helsinki amendments. The institutional review board approved this study (reference number: 201906082RINB) and waived the requirement for informed consent.

**Population Health Research Capsule**

What do we already know about this issue?

*The return of spontaneous circulation after cardiac arrest (RACA) score was developed to estimate the probability of RACA*.

What was the research question?

*Can end-tidal carbon dioxide (EtCO* *_2_* *), a quality indicator of cardiopulmonary resuscitation, be used to improve the RACA score?*

What was the major finding of the study?

*The EtCO_2_ + RACA score (AUC 0.82, 95% CI 0.77*–*0.88) outperformed the original RACA score (AUC 0.7, 95% CI 0.65*–*0.78; P < 0.001)*.

How does this improve population health?

*The EtCO_2_ + RACA score may help allocate medical resources in emergency departments during resuscitation of out-of-hospital cardiac arrest*.

### Study Setting

The NTUH Hospital has 2,600 beds, including 220 beds in intensive care units, and there are approximately 100,000 patient visits to the NTUH ED each year. For OHCA, CPR is performed according to the resuscitation guidelines.^9^,^10^ Furthermore, since 2013 ED personnel who may be involved in the resuscitation of OHCA, including clinicians and nursing staff, have received a specialised training course of the Advanced Cardiac Life Support (ACLS) teamwork model.^12^,^13^ This training model addresses both CPR techniques and non-technical skills.^14^,^15^ Any interventions performed during CPR are timestamped by nurses using a specially designed tablet app. The cardiac rhythm and EtCO_2_ are recorded every two minutes during pulse checking and then uploaded to the electronic health record. The EtCO_2_ is monitored by devices attached to the supraglottic airway (SGA) or endotracheal tube (ETT).

The decision to insert advanced airways, including SGA or ETT, during CPR is at the discretion of the treating physicians. Nonetheless, in our practice most clinicians tend to insert an ETT as soon as possible because, in the ACLS teamwork model, airway management is assigned to a dedicated squad of clinicians and nurses; thus, inserting an ETT does not influence delivery of high quality CPR. Also, for OHCA patients who never achieve ROSC, CPR would usually be performed for at least 30 minutes, except for those with a documented do-not-resuscitate (DNR) order.

### Study Population

Consecutive OHCA patients resuscitated at the ED of NTUH between January 1 2015–December 31, 2020 were screened. Patients fulfilling the following criteria were eligible for inclusion in the study: 1) non-traumatic arrest; 2) absence of ROSC before ED arrival; 3) absence of documentation of DNR order before CPR; 4) transport by EMS; 5) age ≥18 years; 6) insertion of advanced airways, either SGA or ETT; and 7) availability of EtCO_2_ measurement at least once. If a single patient underwent CPR multiple times, we extracted only the first episode for analysis.

### Data Collection, Variable Definitions, and Outcome Measures

In the NTUH ED registry, OHCA scenarios were recorded per the Utstein template.^16^ We used the following variables for analysis: age; gender; variables derived from the Utstein template; management by EMS and in the ED; measured EtCO_2_ levels; and outcomes.

We calculated the RACA score according to the original formula reported by Gräsner et al^6^ (Supplemental Table 1). Nonetheless, the variables of arrest aetiology and location were not explicitly defined by Gräsner et al.^6^ Hence, we defined these variables as follows: 1) After classifying aetiology into trauma, hypoxia, and intoxication in the RACA score, we excluded traumatic OHCA patients in our registry, while including patients with external causes of asphyxia,^16^ and patients with suspected drug overdose;^16^ and 2) we reclassified the arrest location as used in the RACA score. Nursing home designation included assisted living/nursing home; doctor’s office included primary care clinics; public place included sports/recreation event, street/highway, public building, and educational institution; and medical institution included dialysis clinics.

Finally, the initial cardiac rhythms used in the RACA score were those recorded by EMS at initial contact, which were classified into ventricular fibrillation, pulseless electrical activity (PEA), asystole, and other. Nonetheless, our EMS only categorised the initial cardiac rhythms into shockable and non-shockable rhythms. Therefore, we used the initial rhythms recorded upon ED arrival in computing the RACA score. In the NTUH ED registry, the initial 15 EtCO_2_ measurements were recorded. In the current analysis, we retrieved only the EtCO_2_ measured after insertion of advanced airways. The EtCO_2_ summary parameters were computed accordingly, including initial, maximum, minimum, and average EtCO_2_.

The ROSC was specified as the primary outcome and defined as a palpable pulse > 20 seconds, as used by Gräsner et al.^6^ We also reported survival and favourable neurological function at the time of hospital discharge. Favourable neurological function was defined as a score of 1 or 2 on the Cerebral Performance Category scale.^17^

### Sample Size

Because of the retrospective nature of this study, the number of eligible patients during the study period determined the final sample size. We temporally split the final cohort into a derivation cohort and a validation cohort with the ratio of patient numbers being 70% vs 30%.

### Statistical Analysis

Categorical variables are presented as counts with proportions, and continuous variables are presented as medians with interquartile ranges (IQR). We examined categorical variables by chi-square test, while continuous variables were compared by the Wilcoxon rank-sum test.

In the derivation cohort, we calculated the odds ratio (OR) as the outcome measure. We only tested the two predetermined variables, RACA score and minimum EtCO_2_ in the multivariable logistic regression analyses to estimate their association with the primary outcome. We employed generalised additive models (GAM)^18^ to explore non-linear effects of the RACA score or minimum EtCO_2_ on the primary outcome and to identify the optimal cut-off points to transform these variables into categorical variables, if necessary. Since we did not know whether there would be confounding effects or multicollinearity between the RACA score and minimum EtCO_2_, we still conducted formal, stepwise, variable selection procedure with iterations to derive the final prediction model. We defined the significance levels for entry and to stay at *P* = 0.15. We derived the final prediction model by excluding non-significant variables sequentially until all regression coefficients were significant.

In the validation cohort, we assessed the discriminative performance of the derived model by area under the receiver operating characteristic (ROC) curve (AUC). We evaluated model calibration by the Hosmer–Lemeshow goodness-of-fit test and a calibration plot to compare predicted ROSC probabilities with the observed ROSC rates. We compared the AUCs of the EtCO_2_ + RACA and RACA scores by the DeLong test of correlated ROC curves.^19^ We used R 4.1.1 software (R Foundation for Statistical Computing, Vienna, Austria) to analyse the data. A two-tailed *P*-value < 0.05 was considered significant.

## RESULTS

The patient selection procedure resulted in the final cohort of 758 patients (Supplemental Figure 1). We temporally split the final cohort on May 1, 2019, because the ratio of patient numbers in the derivation and validation cohorts was the closest to 70% vs 30%. The characteristics of the patients in the final cohort are presented in Table 1, and we made comparisons between the derivation and validation cohorts.

Overall, the median patient age was 71.0 years (IQR 60.0–82.0 years), and 489 patients (64.5%) were male. Only a small proportion of patients suffered from hypoxia- (5.3%, 40) or intoxication-associated (1.3%, 10) OHCA. Most OHCA occurred at home (60.0%, 455 patients). Approximately 46.2% of OHCA (350 patients) was witnessed by bystanders or EMS, and 52.5% of them received bystander CPR (398 patients). The median time interval between call and EMS arrival was 4.0 minutes (min) (IQR 3.0–5.0 min). A total of 527 patients (69.5%) received SGA placement during prehospital CPR, while 735 patients (97.0%) received an ETT during CPR at the ED. Most of the initial cardiac rhythms recorded upon ED arrival were non-shockable rhythms, including PEA (40.1%, 304 patients) and asystole (53.2%, 403 patients). The median available measurements of EtCO_2_ were 8.0 times (IQR: 3.0–12.0 times), with the median minimum EtCO_2_ of 15.5 mm Hg (IQR: 8.0–26.0 mm Hg). The median RACA score was 36.4% (28.9–48.0%), and a total of 393 patients (51.8%) achieved ROSC.

The differences between patients stratified by ROSC in the derivation cohort are shown in Table 2. Significant differences between patients with and without ROSC were noted in approximately half of the variables included in the RACA score, including age ≥80 years, hypoxia, public place, witness by bystander or EMS, PEA, and asystole. Values of all EtCO_2_ summary parameters were significantly higher in patients with than without ROSC. The GAM plots (Supplemental Figure 2) demonstrated a near-linear association between minimum EtCO_2_ and ROSC. Therefore, we analysed minimum EtCO_2_ as a continuous variable without transformation.

**Figure 1:**
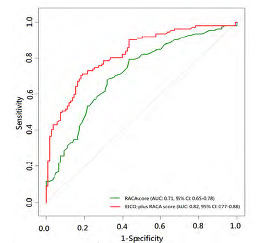
Comparison of receiver operating characteristic curves between the RACA and EtCO2-plus RACA scores. *AUC*, area under ROC curve; *EtCO2*, end-tidal carbon dioxide; *RACA*, return of spontaneous circulation after cardiac arrest.

**Figure 2:**
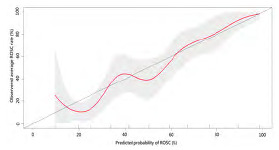
Calibration curve when validating the EtCO2 + RACA score for probability of return of spontaneous circulation (in the validation cohort. The ticks on the X-axis separate the validation cohort into 10 equal patient numbers of subgroups. Red curve, calibration curve; grey area, 95% CI. *EtCO2*, end-tidal carbon dioxide; *ROSC*, return of spontaneous circulation; *RACA*, return of spontaneous circulation after cardiac arrest.

After stepwise variable selection, the final EtCO_2_ + RACA score resulted in good discriminative performance in the derivation cohort (AUC 0.80, 95% CI 0.76–0.83; Table 3) (online EtCO_2_ + RACA score calculator: https://chou2.chou-tw.com/index.php/etco2/). In the validation cohort, the EtCO_2_ +RACA score also demonstrated good discriminative performance (AUC 0.82, 95% CI 0.77–0.88), significantly outperforming the RACA score (AUC 0.71, 95% CI 0.65–0.78) (DeLong test: *P* < 0.001) (Figure 1). Finally, the calibration plot (Figure 2) indicated that EtCO_2_ + RACA score was calibrated well in the validation cohort (Hosmer–Lemeshow test: *P* = 0.33).

## DISCUSSION

### Main Findings

Based on the RACA score, we developed and validated a logistic regression model, the EtCO_2_ + RACA score, to facilitate estimating ROSC probability of OHCA patients transported to EDs for continuing CPR. By adding minimum EtCO_2_ to the original RACA score, the discriminative performance of the EtCO_2_ + RACA score significantly outperformed the original RACA score. By combining a baseline risk-stratifying score and a CPR quality indicator, the EtCO_2_ + RACA score may assist EPs in decision-making regarding OHCA management.

### Comparisons with Previous Studies

For OHCA patients, most prediction models were designed for those who had achieved ROSC,^20^ with only a few models available for patients who were still undergoing CPR. By using registry data, Baldi et al^21^ used Utstein-based (UB) variables to develop the UB-ROSC score for predicting the probability of survival to hospital admission of an OHCA victim with AUCs above 0.77. Also, according to the Utstein-based variables recorded in the PAROS registry, Liu et al^22^ employed machine-learning to derive the prehospital (P-ROSC) score to predict the individualized probability of P-ROSC^23^ with an AUC of 0.806. The RACA score^6^ was originally developed for predicting ROSC after OHCA, using readily available factors when EMS personnel arrive at the scene. With slight differences in definitions, these RACA score-related variables were similar to the Utstein-based ones used by UB-ROSC^21^ and P-ROSC, 22 suggesting that these variables might be employed to make individualized predictions. In the validation cohort of the original study by Gräsner et al,^6^ the RACA score achieved a fair discriminative performance (AUC 0.73).

The RACA score has been validated in many regions, including Finland (AUC 0.71),^24^ Asia (AUC 0.74),^25^ Italy, and Switzerland (AUC 0.76),^26^ as well as in our validation cohort (AUC 0.71), suggesting that the RACA score is generalisable and applicable to a wide range of diverse populations. Our validation cohort had a higher proportion of non-shockable rhythms above 90% (PEA: 39.0%, asystole: 54.4%), compared with the validation cohort of the Gräsner et al^6^ study (PEA: 15.0%, asystole: 46.6%). Despite the substantial difference in the presenting rhythms, the discriminative performance of the RACA score in our validation cohort was similar to that in previous studies (AUC 0.71, Figure 1). Therefore, we adopted the RACA score in our study to calculate the baseline probability of ROSC.

The ROSC rate (51.8%) of our total cohort was slightly higher than the ROSC rate (43.3%) reported by Gräsner et al,^6^ despite the fact that the proportions of non-shockable rhythms were higher in our study. When using the EMS registry of our city, the Chiang et al study^27^ reported the proportion of survival or favourable neurological outcome at hospital discharge among OHCA patients receiving ETT or SGA was 7.2% and 3.1%, respectively, similar to our results (survival: 10.3%, favourable neurological outcome: 5.4%). Chiang et al^27^ also noted high proportions of non-shockable rhythms (89.2%) and sustained ROSC of ≥2 hours (26.6%) among these patients. Since ROSC was defined as a palpable pulse lasting longer than 20 seconds^6^ in our study, the seemingly high ROSC rate may be reasonable.

The RACA score tended to underestimate the ROSC probability in our cohort, with predicted vs observed ROSC rate being 36.4% vs 51.8% (Table 1). This miscalibration may have occurred because in the original study in which the RACA score was developed,^6^ the arrest aetiology and location were not classified according to the Utstein template^16^ and needed to be reclassified in our study retrospectively. Furthermore, the component variables of the RACA score, such as age or arrest location, were all baseline variables and unmodifiable during CPR. These baseline variables may not reflect the highly dynamic nature of CPR. That is, if the ROSC probability predicted by the RACA score was low, the outcomes could still be improved by high quality CPR; in contrast, even if the RACA score predicted a high ROSC probability, the outcomes may still be compromised if CPR quality was poor. Hence, to make individualised prognostication during CPR, variables specific to the patient and resuscitation process such as EtCO_2_ may be more helpful.

### Interpretation of Current Studies

The systematic review by Paiva et al^28^ indicated that EtCO_2_ was associated with ROSC probability, likely because EtCO_2_ could reflect CPR quality. Nonetheless, the optimal parameter for EtCO_2_ being a prognostic factor is still under debate.^28^ For example, the so-called “initial” EtCO_2_ may not be truly measured at the initial stage of CPR since an ETT may be inserted at a later stage of CPR. Also, the maximum EtCO_2_ may be confusing since it did not account for the influence of measurement timing, ie, whether the predictive value of a maximum EtCO_2_ measured at the early stage of CPR was the same as that measured at the late stage of CPR. Average EtCO_2_ was also a frequently reported parameter. Despite its convenience in summarising the overall measured EtCO_2_, average EtCO_2_ could not differentiate between different EtCO_2_ trends. While ascending and descending EtCO_2_ trends may have similar average EtCO_2,_ their prognoses may be very different.^29^ Furthermore, average EtCO_2_ is not very practical in clinical application since clinicians may not be able to compute the average EtCO_2_ in real time. Lastly, the most promising parameter in predicting ROSC may be the minimum EtCO_2_ since this parameter may reflect the minimum CPR quality achieved by rescuers.

The higher the minimum EtCO_2_, the higher the ROSC probability would be (Supplemental Figure 2). Similarly, the systematic review of Paiva et al^28^ indicated that EtCO_2_ ≥ 20 mm Hg was a stronger predictor for ROSC than EtCO_2_ ≥10 mm Hg. An abrupt rise of EtCO_2_ over 40 mm Hg was suggested to be the first sign of ROSC.^28^ For minimum EtCO_2_ ≤40 mm Hg, the higher EtCO_2_ may indicate higher CPR-generated cardiac output and better CPR quality. In contrast, for minimum EtCO_2_ >40 mm Hg, the high EtCO_2_ may simply suggest that the cardiac arrest was caused by asphyxia or hypercapnic respiratory failure prior to collapse rather than augmented cardiac output.^30^

Our research has several advantages over previous studies^28^ in investigating EtCO_2_ as an outcome predictor. First, as one part of the ACLS teamwork model,^12^,^13^ the EtCO_2_ is routinely recorded in our ED. Second, the clinical practice is consistent across different clinicians who treat OHCA in our ED’s resuscitation bay. As shown in Table 1, the proportions of ETT use in the ED were similarly high in the derivation and validation cohorts. Finally, CPR is usually performed for approximately 30 min in patients who never achieve ROSC (median duration of CPR performed in the ED was 28.5 min, Table 1), which would not be shortened simply because of clinicians’ perception of poor prognosis for the patient. This practice may help circumvent the “bias of self-fulfilling prophecy”^31^ since EtCO_2_ has been proposed as a variable in the termination of resuscitation rule.^9^

### Future Applications

In most Asian countries, EMS personnel are usually not legally allowed to pronounce death^2^ and must transfer OHCA patients to EDs despite most interventions of Advanced Life Support being able to be performed in prehospital resuscitation. The EPs resuscitating EMS-transported patients are faced with the problem of whether resuscitation should be continued as set up by EMS personnel or further invasive interventions should be implemented. Advanced therapeutics, such as extracorporeal CPR^5^ or resuscitative endovascular balloon occlusion of the aorta,^32^ are being tested and may be applicable in the near future for OHCA. Nonetheless, these interventions may only be beneficial in a certain group of patients. A prediction model like the EtCO_2_ + RACA score may assist EPs in determining whether the interventions should be upgraded for an individual patient. It should be emphasized that like other scoring systems predicting ROSC for OHCA patients,^21^,^22^ the EtCO_2_ + RACA score is not intended to terminate CPR for OHCA patients. With the assistance of advanced technology, the EtCO_2_ + RACA score may be computed instantaneously along with the real-time updated minimum EtCO_2_ during CPR. Further studies are needed to validate the EtCO_2_ + RACA score and explore the possibility of integrating the prediction model, internet-based devices, and the resuscitation process.

## LIMITATIONS

First, the analysed EtCO_2_ dataset was derived from a prospectively collected database of a single centre with a specialised training model for CPR. The median of the minimum EtCO_2_ was higher than 10 mm Hg (Table 1), revealing the high CPR quality achieved by the ACLS teamwork model. Further studies are needed to investigate the generalisability of our results. Second, as suggested by the TRIPOD statement,^11^ the temporally split derivation and validation cohorts allowed non-random variation between the two cohorts, and this kind of validation could be considered an intermediary between internal and external validation.

Even the number of measurements of EtCO_2_ was significantly higher in the validation than the derivation cohort (Table 1), suggesting that the ACLS teamwork model matured with time. Despite this advantage in the validation procedure, further external validation of the EtCO_2_ + RACA score in other communities is needed because this model was developed from a single medical centre. Finally, we only used EtCO_2_ recorded in the ED to develop the EtCO_2_ + RACA score. Because of the limitations in the facility and human resources, the EtCO_2_ values were not recorded at regular time intervals in the field and during transport. Future studies are needed to test whether the EtCO_2_ values measured by EMT during CPR could further improve the performance of the EtCO_2_ + RACA score.

## CONCLUSION

The derived EtCO_2_ +RACA score may be used to assist emergency physicians in estimating the probability of return of spontaneous circulation for EMS-transported patients with out-of-hospital cardiac arrest. By adding a CPR quality indicator, minimum EtCO_2_, to the well-validated RACA score, the EtCO_2_ + RACA score achieved good discriminative performance. The EtCO_2_ + RACA score may facilitate the decision-making process regarding allocations of medical resources in EDs for OHCA resuscitation. Nonetheless, it should be emphasized that the EtCO_2_ + RACA score is not intended to terminate CPR. Further validation by external datasets is warranted to ensure the generalizability of the EtCO_2_ + RACA score.

## Supplementary Information







## Figures and Tables

**Table 1:** Characteristics of all included patients during the study period.

Variables	Total cohort (N = 758)	Derivation cohort (n = 530)	Validation cohort (n = 228)	*P*-value
Age, year	71.0 (60.0–82.0)	73.0 (60.0–83.0)	68.0 (59.0–79.0)	0.005
**Age ≥ 80 years**	227 (29.9)	173 (32.6)	54 (23.7)	0.01
**Male**, n	489 (64.5)	335 (63.2)	154 (67.5)	0.25
Arrest etiology, n				
**Hypoxia**	40 (5.3)	33 (6.2)	7 (3.1)	0.07
**Intoxication**	10 (1.3)	7 (1.3)	3 (1.3)	0.99
Arrest location, n				
At home	455 (60.0)	325 (61.3)	130 (57.0)	0.27
**Nursing home**	28 (3.7)	25 (4.7)	3 (1.3)	0.02
**Doctor’s office**	13 (1.7)	8 (1.5)	5 (2.2)	0.51
**Public place**	172 (22.7)	109 (20.6)	63 (27.6)	0.03
**Medical institution**	6 (0.8)	6 (1.1)	0 (0)	0.11
Witness status, n				
**Witness by bystander**	313 (41.3)	214 (40.4)	99 (43.4)	0.44
**Witness by EMS**	58 (7.7)	48 (9.1)	10 (4.4)	0.03
Witness by bystander or EMS	350 (46.2)	242 (45.7)	108 (47.4)	0.67
**Bystander CPR**, n	398 (52.5)	265 (50.0)	133 (58.3)	0.04
EMS management				
**Call to EMS arrival**, minutes	4.0 (3.0–5.0)	4.0 (3.0–5.0)	4.0 (3.0–6.0)	0.01
Prehospital SGA use, n	527 (69.5)	372 (70.2)	155 (68.0)	0.55
Prehospital ETT use, n	54 (7.1)	37 (7.0)	17 (7.5)	0.82
Prehospital epinephrine use, n	187 (24.7)	119 (22.5)	68 (29.8)	0.03
Prehospital defibrillation, n	150 (19.8)	96 (18.1)	96 (23.7)	0.08
Prehospital CPR duration, minutes	17.0 (13.0–21.0)	16.0 (12.0–20.0)	19.0 (16.0–23.0)	<0.001
ED management				
Initial cardiac rhythms at ED arrival, n				
Shockable rhythms	51 (6.7)	36 (6.8)	15 (6.6)	0.91
**PEA**	304 (40.1)	215 (40.6)	89 (39.0)	0.69
**Asystole**	403 (53.2)	279 (52.6)	124 (54.4)	0.66
ED SGA use, n	526 (69.4)	371 (70.0)	155 (68.0)	0.58
ED ETT use, n	735 (97.0)	510 (96.2)	225 (98.7)	0.07
Available measurements of EtCO_2_, times	8.0 (3.0–12.0)	6.0 (3.0–11.0)	11.0 (4.0–13.0)	<0.001
EtCO_2_ summary parameters, mm Hg				
Initial	26.0 (16.0–41.0)	25.0 (16.0–40.0)	27.0 (15.5–42.5)	0.40
Maximum	39.0 (25.0–54.0)	37.5 (24.0–52.0)	41.0 (25.5–56.0)	0.19
Minimum	15.5 (8.0–26.0)	16.0 (9.0–26.0)	15.0 (7.0–26.0)	0.57
Average	26.4 (16.0–38.3)	25.7 (16.5–37.8)	27.4 (15.3–39.1)	0.66
ED CPR duration, min	28.5 (12.0–32.0)	26.0 (12.0–32.0)	30.0 (13.0–31.0)	0.38
ROSC probability predicted by RACA score, %	36.4 (28.9–48.0)	36.4 (28.5–48.5)	36.8 (29.3–47.5)	0.69
Outcome, n				
ROSC	393 (51.8)	281 (53.0)	112 (49.1)	0.33
Survival to hospital discharge	78 (10.3)	53 (10.0)	25 (11.0)	0.69
Favorable neurological outcome at hospital discharge	41 (5.4)	28 (5.3)	13 (5.7)	0.82

Data are presented as median (interquartile range) or counts (proportion). Bold-typed variables represent those used in the RACA score.

*EMS*, emergency medical service; *CPR*, cardiopulmonary resuscitation; *SGA*, supraglottic airway; *ETT*, endotracheal tube; *ED*, emergency department; *EtCO_2_*, end-tidal carbon dioxide; *mm Hg*, millimeters of mercury; *PEA*, pulseless electrical activity.

*ROSC*, return of spontaneous circulation; *RACA*, ROSC after cardiac arrest.

**Table 2:** Characteristics of patients in derivation cohort stratified by return of spontaneous circulation.

Variables	Derivation cohort (n = 530)	ROSC (n = 281)	Absence of ROSC (n = 249)	*P*-value
Age, year	73.0 (60.0–83.0)	70.0 (60.0–82.3)	75.0 (61.0–84.0)	0.05
**Age ≥ 80 years**	173 (32.6)	80 (28.5)	93 (37.3)	0.03
**Male**, n	335 (63.2)	188 (66.9)	147 (59.0)	0.06
Arrest etiology, n				
**Hypoxia**	33 (6.2)	25 (8.9)	8 (3.2)	0.007
**Intoxication**	7 (1.3)	4 (2.5)	3 (1.2)	0.83
Arrest location, n				
At home	325 (61.3)	148 (52.7)	177 (71.1)	<0.001
**Nursing home**	25 (4.7)	10 (3.6)	15 (6.0)	0.18
**Doctor’s office**	8 (1.5)	6 (2.1)	2 (0.8)	0.21
**Public place**	109 (20.6)	74 (26.3)	35 (14.1)	<0.001
**Medical institution**	6 (1.1)	5 (1.8)	1 (0.4)	0.13
Witness status, n				
**Witness by bystander**	214 (40.4)	141 (50.2)	73 (29.3)	<0.001
**Witness by EMS**	48 (9.1)	35 (12.5)	13 (5.2)	0.004
Witness by bystander or EMS	242 (45.7)	161 (57.3)	81 (32.5)	<0.001
**Bystander CPR**, n	265 (50.0)	142 (50.5)	123 (49.4)	0.79
EMS management				
**Call to EMS arrival**, minutes	4.0 (3.0–5.0)	4.0 (3.0–5.0)	4.0 (3.0–5.0)	0.15
Prehospital SGA use, n	372 (70.2)	189 (67.3)	183 (73.5)	0.12
Prehospital ETT use, n	37 (7.0)	15 (5.3)	22 (8.8)	0.12
Prehospital epinephrine use, n	119 (22.5)	56 (19.9)	63 (25.3)	0.14
Prehospital defibrillation, n	96 (18.1)	68 (24.2)	28 (11.2)	<0.001
Prehospital CPR duration, minutes	16.0 (12.0–20.0)	16.0 (11.0–19.0)	17.0 (13.0–20.0)	0.005
ED management				
Initial cardiac rhythms at ED arrival, n				
Shockable rhythms	36 (6.8)	30 (10.7)	6 (2.4)	<0.001
**PEA**	215 (40.6)	132 (47.0)	83 (33.3)	0.001
**Asystole**	279 (52.6)	119 (42.3)	160 (64.3)	<0.001
ED SGA use, n	371 (70.0)	189 (67.3)	182 (73.1)	0.14
ED ETT use, n	510 (96.2)	271 (96.4)	239 (96.0)	0.78
Available measurements of EtCO2, times	6.0 (3.0–11.0)	4.0 (2.0–8.0)	10.0 (5.0–13.0)	<0.001
EtCO2 summary parameters, mm Hg				
Initial	25.0 (16.0–40.0)	31.0 (21.0–45.0)	21.0 (12.0–30.3)	<0.001
Maximum	37.5 (24.0–52.0)	43.0 (30.0–57.0)	31.0 (18.0–48.0)	<0.001
Minimum	16.0 (9.0–26.0)	22.0 (15.0–32.3)	11.0 (5.0–18.0)	<0.001
Average	25.7 (16.5–37.8)	30.5 (22.9–43.1)	19.9 (11.2–29.1)	<0.001
ED CPR duration, minutes	26.0 (12.0–32.0)	14.0 (9.0–28.0)	31.0 (27.0–34.0)	<0.001
ROSC probability predicted by RACA score, %	36.4 (28.5–48.5)	41.1 (31.4–54.5)	31.9 (26.1–39.8)	<0.001

Data are presented as median (interquartile range) or counts (proportion). Bold-typed variables represent those used in the RACA score.

*EMS*, emergency medical service; *CPR*, cardiopulmonary resuscitation; *SGA*, supraglottic airway.

*ETT*, endotracheal tube; *ED*, emergency department; *EtCO_2_*, end-tidal carbon dioxide; *mm Hg*, millimeters of mercury; *PEA*, pulseless electrical activity.

**Table 3:** The EtCO_2_-plus RACA score

Intercept and predictors	β coefficient	Odds ratio (95% CI)	*P*-value
Intercept	−3.34284		
RACA score	5.76509	318.97 (62.63–1624.36)	<0.001
Minimum EtCO_2_, mm Hg	0.06721	1.07 (1.05–1.09)	<0.001

The predicted probability of return of spontaneous circulation (ROSC) can be calculated using the following formula: probability of ROSC = 1/{1 + exp[−(−3.34284 + RACA score × 5.76509 + minimum EtCO_2_ × 0.06721)]}.

*EtCO_2_*, end-tidal carbon dioxide; *RACA*, return of spontaneous circulation after cardiac arrest.
