# Supplementary figures and images for: End-tidal Carbon Dioxide + Return of Spontaneous Circulation After Cardiac Arrest (RACA) Score to Predict Outcomes After Out-of-hospital Cardiac Arrest
Source: West J Emerg Med. 2023 Apr 4;24(3):605–14. doi: 10.5811/westjem.59005 (PMC10284512; doi:10.5811/westjem.59005)

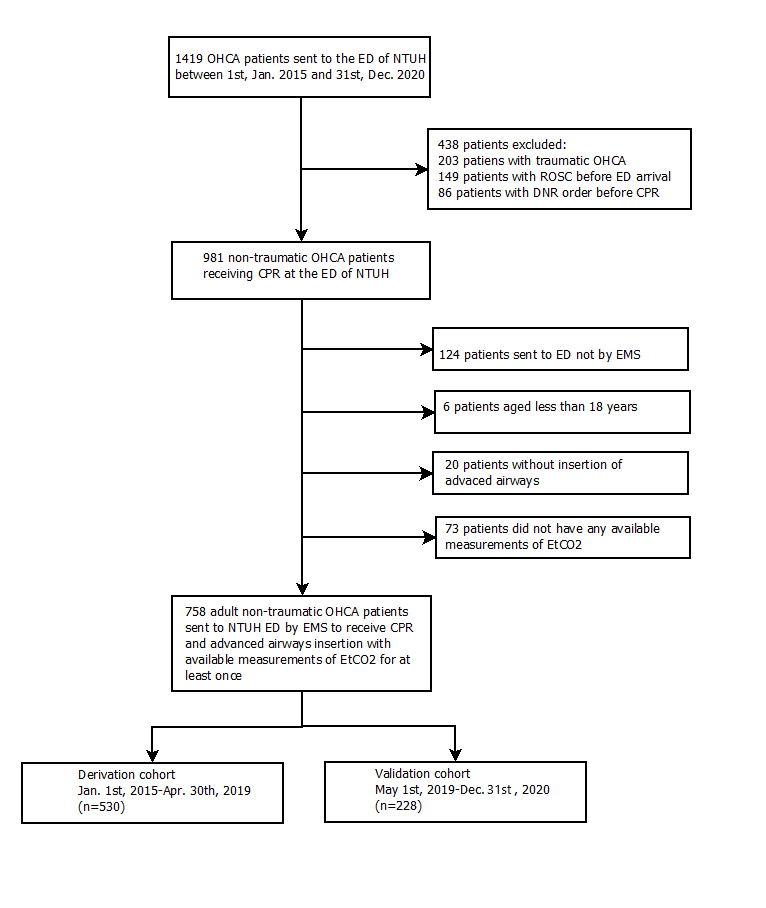

Supplement: Supplementary file 1 [file wjem-24-605-s001.tif]

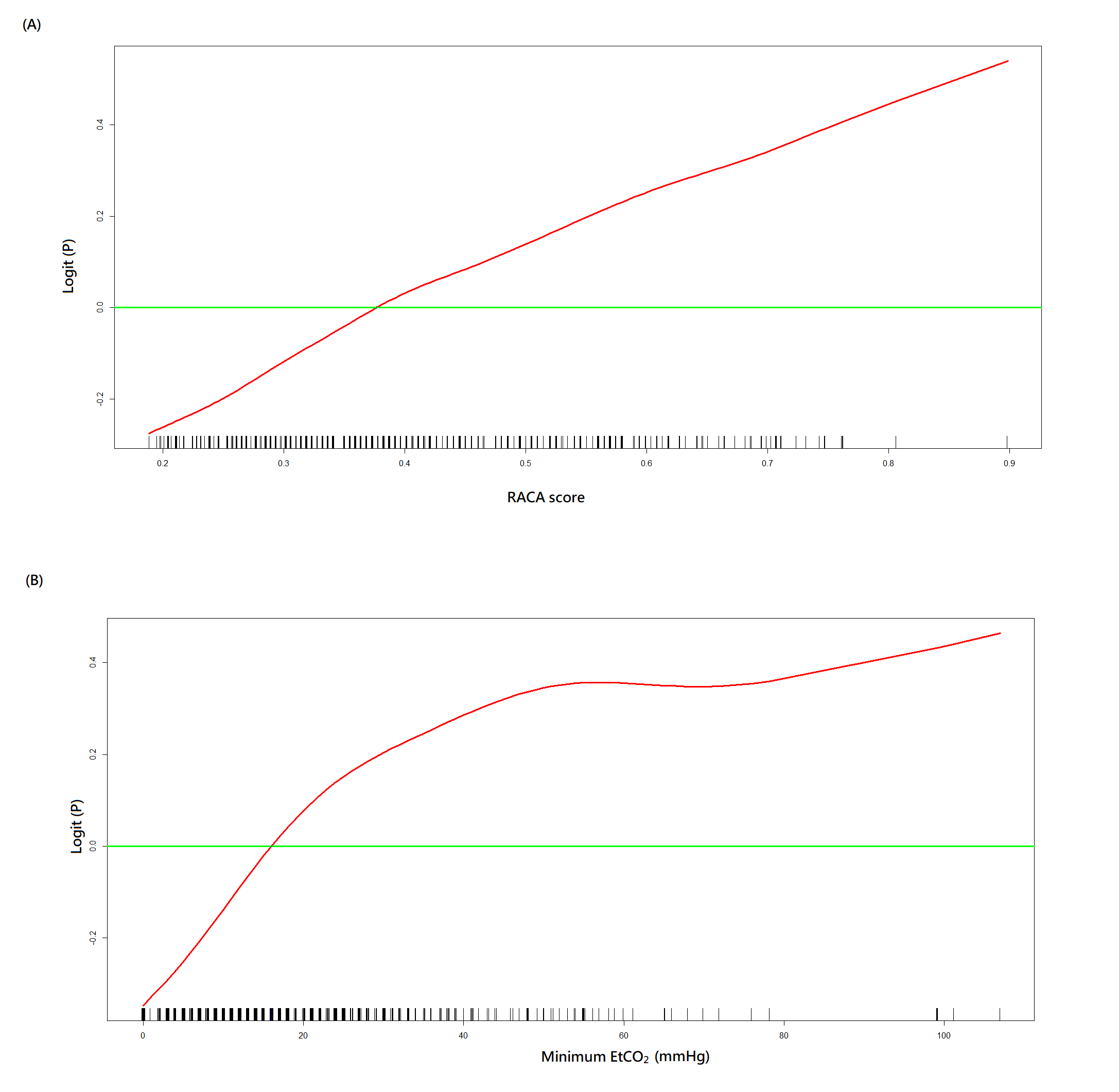

Supplement: Supplementary file 2 [file wjem-24-605-s002.tif]
