# Supplementary material for: End-tidal Carbon Dioxide + Return of Spontaneous Circulation After Cardiac Arrest (RACA) Score to Predict Outcomes After Out-of-hospital Cardiac Arrest
Source: West J Emerg Med. 2023 Apr 4;24(3):605–14. doi: 10.5811/westjem.59005 (PMC10284512; doi:10.5811/westjem.59005)
Supplement: Supplementary file 3 [file wjem-24-605-s003.docx]

**Supplemental Table 1.** Formulae for RACA score.

| Score | Formula |
| --- | --- |
| RACA score | X = 0.3 (constant) + (−0.2 × male) + (−0.2 × age ≥ 80 years) + (−0.6 × trauma) + (0.7 × hypoxia) + (0.5 × intoxication) + (0.6 × witnessed by lay people) + (0.5 × witnessed by professionals) + (−0.3 × nursing home) + (1.2 × doctor’s office) + (0.3 × public place) + (0.5 × medical institution) + (−0.8 × PEA) + (−1.1 × asystole) + (0.2 × bystander CPR) + (−0.04 × minutes until EMS arrival)  Probability of ROSC = 1/(1+e^−x^) |

Equation of the RACA (ROSC after cardiac arrest) score. Score value X will be transformed into a probability of return of spontaneous circulation (ROSC) by logistic function. PEA, pulseless electrical activity; CPR, cardiopulmonary resuscitation; EMS, emergency medical services.
